# High-Intensity Functional Training for Older Adults with Mobility Disabilities: A Feasibility Pilot Study

**DOI:** 10.3390/healthcare14030349

**Published:** 2026-01-30

**Authors:** Lyndsie M. Koon, Joseph E. Donnelly, Jacob J. Sosnoff, Abbas Tabatabaei, Joseph R. Sherman, Anna M. Rice, Morgan Means, Reed Handlery, Kaci Handlery

**Affiliations:** 1 Life Span Institute, University of Kansas, Lawrence, KS 66045, USA; 2 Division of Physical Activity and Weight Management, Department of Internal Medicine, University of Kansas Medical Center, Kansas City, KS 66160, USA; 3 Department of Physical Therapy, Rehabilitation Science, and Athletic Training, Landon Center on Aging, University of Kansas Medical Center, Kansas City, KS 66160, USA; 4 School of Physical Therapy and Rehabilitation Sciences, Morsani College of Medicine, University of South Florida, Tampa, FL 33612, USA; 5 Arkansas Colleges of Health Education, School of Physical Therapy, Fort Smith, AR 72916, USA

**Keywords:** high-intensity functional training, mobility disability, older adults, adaptive exercise, feasibility, physical function, community-based intervention

## Abstract

Background/Objectives: There is limited empirical evidence on the feasibility of inclusive, community-based exercise programs for older adults with long-term mobility disabilities. This pilot study investigated the feasibility and preliminary effectiveness of a community-based high-intensity functional training (HIFT) intervention. Methods: This single-group pre–post feasibility trial was delivered across four community-based HIFT facilities. Thirteen participants enrolled, and 10 (mean age 69.8 ± 6.7 years; 60% female) completed baseline assessments, two onboarding sessions, and thrice-weekly group-based workouts across 16 weeks. Physical function was assessed using the Canadian Occupational Performance Measure (COPM), Patient-Reported Outcomes Measurement Information System (PROMIS) Physical Function, Modified Falls Efficacy Scale (MFES), and standardized tests of mobility, balance, and strength. Exploratory outcomes included body mass index (BMI), waist circumference, work capacity, and quality of life (QOL). Results: Recruitment, retention, and attendance rates were 38%, 77%, and 58% (80% including make-up sessions), respectively. The intervention was safe and well-tolerated, with one fall-related adverse event. Self-reported functional outcomes demonstrated small to large effects, with large improvements in participant-identified functional activities (*d* = 1.03–1.54) and fall efficacy (*d* = 0.97), and a small effect for standardized physical function (*d* = 0.36) Endurance improved substantially (*d* = 1.01), while mobility, balance, and strength outcomes reflected maintenance or small to moderate gains (*d* = 0.08–0.55). BMI remained stable (*d* = 0.05), work capacity increased with moderate to large effects (*d* = 0.61–1.43), and QOL improved modestly (*d* = 0.20). Exit interviews reinforced high acceptability, highlighting individualized adaptations, supportive trainers, and the group-based context as motivating contextual factors. Conclusions: A community-based HIFT program is feasible and acceptable for older adults with mobility disabilities.

## 1. Introduction

Aging with a mobility disability presents distinct challenges to preventing secondary health conditions, maintaining functional independence, and sustaining quality of life [1,2]. Mobility disabilities become increasingly common with age, affecting 15% of adults ages 65–74 years, 26% of adults ages 75–84 years, and nearly half of those aged ≥85 years [3,4]. Some individuals acquire disability later in life as part of the aging process, while others enter older adulthood already living with a long-term mobility disability [5]. Within those aging with long-term disability, limitations are typically multifactorial, reflecting the interaction between pre-existing impairments and age-related declines in strength, balance, or cardiopulmonary reserve, as well as the onset of musculoskeletal or neurological conditions, sensory impairments, or even medication-related effects [1,6,7,8,9]. Together, these factors elevate the risk for functional decline, increase vulnerability to secondary health conditions, and result in greater support needs.

Exercise participation can improve physical function, mobility, balance, and other health-related outcomes for adults with disabilities [9,10,11], yet recommendations for exercise by healthcare providers are frequently limited to referrals for short-term, outpatient rehabilitation, which fails to provide a sustainable strategy for the delivery of exercise [12]. Community-based programs that facilitate long-term activity engagement beyond the clinical setting are necessary for adults with mobility disabilities [13,14,15,16]. Recent evidence demonstrates that when accessible facilities, trained staff, and individualized programming are provided, community-based exercise programs can improve strength and endurance, and support sustained participation for adults with various mobility disabilities [17]. However, persistent challenges often reduce access for people with mobility disabilities due to inaccessible physical spaces and/or equipment, transportation limitations, or a lack of adaptive support and instruction from experienced trainers [15,18,19,20,21,22]. Despite this early evidence, few studies have evaluated community-based programs that address access barriers and are designed specifically for older adults with mobility disabilities, a population at elevated risk for declining functional capacity [6,8,23] and whose exercise needs may differ from those of younger mobility-limited adults or nondisabled older adults.

High-Intensity Functional Training (HIFT) offers a promising, community-based approach to meet the exercise needs of adults with mobility disabilities. HIFT emphasizes constantly varied, functional movements—such as squatting, lifting, pushing, and transferring—that mirror activities of daily living [24,25]. Emerging evidence supports the feasibility, safety, and preliminary effectiveness of HIFT for adults with mobility disabilities and neurologic conditions, including spinal cord injury, Parkinson’s disease, and mixed mobility-limited populations [26,27,28], as well as among older adults without disability [29]. These studies report high feasibility and initial evidence of effectiveness on functional outcomes, including mobility, strength, balance, and fitness—suggesting that community-delivered HIFT may provide a sustainable pathway for maintaining physical function among individuals aging with disabilities.

To date, no studies have evaluated community-based HIFT specifically among older adults with mobility disabilities. To address this gap, the present study evaluated the feasibility and preliminary effects of a 16-week community-based HIFT intervention for older adults with mobility disabilities. Outcomes included recruitment, retention, and attendance; preliminary safety and acceptability; and self-report and performance-based functional assessments to determine whether this scalable delivery model represents a viable strategy for supporting health among older adults with mobility disabilities.

## 2. Materials & Method

### 2.1. Design

This was a single-group, pre–post pilot clinical trial with assessments at baseline and post-intervention. This clinical trial was approved by the University of Kansas Medical Center Institutional Review Board (STUDY00150386) and registered at ClinicalTrial.gov (NTC07283510).

### 2.2. Recruitment

Recruitment occurred through community organizations (e.g., Centers for Independent Living), physician offices, healthcare providers, and social media advertisements targeting older adults with mobility disabilities in the greater Kansas City region from August 2023 through December 2023. Interested individuals completed an initial screening survey to determine eligibility. Adults who were aged 60 years and older with a self-reported permanent mobility disability of at least one year in duration, and were ambulatory, defined as the ability to walk independently or with an assistive device for short distances to complete functional performance-based assessments, were eligible for the study. Participants were excluded if they were not granted approval for study participation by their healthcare provider or were currently engaged in a structured exercise program. The target sample size (N = 13) was determined based on feasibility parameters, including the scope of the pilot study, available funding, and the time allotted to complete the intervention and assessments. Participants received $100 total for completing pre- and post-intervention assessments, and HIFT membership costs were covered for the 16 weeks. Written informed consent was obtained from all participants before enrollment.

### 2.3. Intervention

The intervention was a 16-week community-based HIFT program, delivered at four community facilities in partnership with Adaptive Athletes in Motion, a nonprofit organization that has provided adaptive HIFT programs for individuals with disabilities in the greater Kansas City area for over 10 years. This partnership provided an experienced trainer with a CrossFit Level 3 and Adaptive and Inclusive Training (AIT) certification through the Adaptive Training Academy [30] who developed and disseminated adaptive programming; trained coaches at other sites in onboarding procedures, intervention implementation, and tracking log use; and delivered hands-on guidance in adaptive and inclusive strategies to ensure successful program delivery across sites. Participants enrolled at the site most convenient to them and could attend sessions at other participating facilities as needed, reflecting real-world delivery of community-based HIFT.

All participating facilities provided accessible training environments, including open floor plans, adaptable equipment, and space to accommodate assistive devices, seated exercise, and individualized movement modifications. Each facility offered four sessions per week, and participants were asked to attend three sessions per week for 16 weeks. Sessions were delivered in a group-based format, and programming emphasized functional, multi-joint movements, adapted to address individual functional limitations, and scaled to each participant’s preferences and ability levels [24,25]. All sessions were led by trainers who held a CrossFit Level 1 or higher and an AIT certification. Lead trainers at each site also had a minimum of two years of experience coaching adaptive HIFT for people with disabilities.

Movements incorporated body weight, resistance bands, dumbbells, barbells, kettlebells, and other accessible equipment. Trainers provided multiple adaptation options to ensure safe and effective engagement across a range of functional abilities, including modifications for seated and standing performance and assistive device use. Common movements included squats, push-ups, overhead presses, and deadlifts, as well as rowing, biking, and skiing. Adaptations were individualized and included seated or box-supported squats; wall push-ups to reduce shoulder strain; single-arm or banded presses; and elevated or partial-range deadlifts from blocks or boxes. Participants were also able to perform movements while seated as needed. Trainers provided verbal cues, ensured stable surfaces, and offered spotting or assistive contact as needed to reduce fall risk and ensure safety during all sessions. The 60 min sessions maintained a 5:1 participant-to-trainer ratio, with graduate and undergraduate student volunteers from health-related disciplines (e.g., exercise science, occupational therapy, physical therapy) providing additional support. Following a structured training session led by the primary trainer, volunteers assisted with equipment setup and transitions, monitored participant safety, and provided verbal cues and reminders as needed under trainer supervision. 

Before beginning the intervention, participants completed two individual onboarding sessions with the primary trainer at their selected facility. These sessions introduced the class structure and equipment; explained technique, scaling options, and seated/standing adaptations; and addressed considerations for assistive devices (e.g., safe transitions, movement modifications, and equipment setup) tailored to participants’ mobility needs. Safety procedures were emphasized throughout, with trainers collaborating with participants to identify personalized modifications to support safe and effective engagement.

HIFT sessions followed a consistent structure, beginning with a warm-up (5–10 min of mobility and dynamic stretching), then a skill or strength segment focusing on a specific movement or muscle group, followed by a workout of the day (WOD) lasting 10–25 min, and concluding with a cool-down that included stretching and guided recovery. The WOD consisted of varied formats, including as many rounds or repetitions as possible (AMRAP), rounds for time (RFT), and every minute on the minute (EMOM), with adaptations and scaling to modify load, range of motion, or equipment to accommodate preferences, abilities, and limitations (see Table 1, for example, WODs). Following each session, trainers completed a brief training log to record participant attendance, adaptations used, WOD performance results, participant-reported issues (e.g., fatigue, pain), and perceived session intensity (described below).

Perceived exertion was selected as the primary intensity monitoring method to reflect real-world community-based implementation and to accommodate variability in exercise response among older adults with mobility disabilities. Intensity was prescribed and monitored using the Borg CR-10 scale for Rating of Perceived Exertion (RPE) [31], a valid and reliable tool for assessing intensity during HIFT [32,33]. Participants were instructed to self-regulate effort based on comfort, ability, and health status, progressing from light to moderate intensity (RPE 1–3) during the initial weeks to moderate to vigorous intensity (RPE 4–8) throughout the remainder of the intervention. Trainers recorded each participant’s overall session-level RPE immediately after the cool-down, providing a practical indicator of internal training load and perceived exertion across sessions [34]. Exercise progression followed a standardized HIFT framework [25,35] and was individualized based on participant tolerance, movement quality, and RPE.

Weekly programming plans were distributed to site coaches, who completed logs after each session documenting attendance, adaptations provided, participant WOD performance, and any participant-reported concerns (e.g., pain, fatigue) (See Table 2). These logs were used to monitor adherence to the planned session structure and note any individualized adaptations implemented. A member of the research team attended one class per week, rotating across sites, to observe safety practices, session flow, trainer-participant ratios, and individualized adaptations. The research team and lead trainer reviewed completed logs weekly and provided guidance to site trainers as needed to maintain consistency in program delivery across sites. Because maintaining participation was a key feasibility objective, the research team monitored attendance weekly. When a participant missed two or more planned sessions within a week, a research team member first contacted the site lead trainer to gather information about the absence. If necessary, the research team contacted the participant to identify barriers (e.g., travel, medical appointments) and to discuss plans to improve attendance in subsequent weeks. Across the intervention period, session observations and training logs indicated consistent adherence to the intended structure of the HIFT sessions and individualized adaptation strategies across sites.

### 2.4. Measures

Demographics & Feasibility. Participant demographic characteristics (e.g., age, sex, race/ethnicity) and disability-related descriptors (e.g., self-reported primary disability diagnosis or condition such as neurological, musculoskeletal, etc., assistive device use) were collected through a pre-intervention survey administered to all individuals who provided informed consent. Recruitment rate was defined as the proportion of individuals who were screened and subsequently enrolled in the study, whereas the retention rate was defined as the proportion of enrolled participants who completed the full 16-week intervention and post-intervention assessments. Attendance was calculated as both the (i) total number of sessions attended out of 48 prescribed sessions and (ii) average number of sessions attended per week. Feasibility outcomes were evaluated against established pilot feasibility thresholds (e.g., ≥70% recruitment, ≥80% retention, and ≥75% attendance) [36,37]. Adverse events were documented if they occurred during a HIFT session or outside of the program but impacted participation. Semi-structured exit interviews captured participant experiences and perceptions of program acceptability, perceived health benefits, and barriers and facilitators to participation [38].

Physical Function. Self-reported physical function was assessed by the Canadian Occupational Performance Measure (COPM) at baseline and after 16-week intervention. COPM is a valid, reliable, and responsive [39] measure including semi-structured interview which measures self-perceived performance and satisfaction with performance in daily activities that participants identify as important [40]. At baseline, participants selected up to five occupational activities and then rated each on a 10-point Likert-type scale for performance (*1 = not able to do, 10 = able to do extremely well*) and satisfaction with performance (*1 = not satisfied at all, 10 = extremely satisfied*). Post-intervention, participants repeated the measure. Separate mean scores were calculated for performance and satisfaction, based on the average of participants’ ratings across their selected activities.

Participants also completed the PROMIS Physical Function Computer Adaptive Test (CAT) [41,42], which tailors items to each individual, and thus, not all participants received the same set of questions. Participants rated their ability to perform a range of mobility and daily activities (e.g., walking, lifting, carrying) using a five-point Likert-type response format. The instrument has demonstrated excellent internal consistency reliability across adult populations (Cronbach’s α = 0.92–0.97), as well as strong construct validity and responsiveness [42,43,44], and was scored using the Health Measures Scoring Service, yielding standardized T-scores [45].

Confidence in performing daily activities without falling was measured using the Modified Falls Efficacy Scale (MFES) [46]. The MFES consists of 14 items rated from 0 (*not confident at all*) to 10 (*completely confident*), with higher scores indicating greater fall-related self-efficacy. The MFES demonstrates high internal consistency (Cronbach’s α = 0.95–0.97) and test–retest reliability among older adults [46,47] and individuals with mobility limitations [48,49,50].

Performance-based physical function was evaluated using valid and reliable tests of mobility, balance, and strength in older adults. Assessments were conducted across multiple scheduled visits to minimize participant burden and fatigue. The 2-Minute Step Test, Timed Up and Go, and Five-Times Sit-to-Stand tests were administered during a scheduled assessment visit at participants’ selected community-based training facilities. Additional performance-based assessments, including the 10-Meter Walk Test, balance assessments, and grip strength, were conducted during a separate scheduled visit at the University of Kansas Landon Center on Aging, Mobility and Falls Laboratory. Mobility was evaluated with the 2-Minute Step Test, Timed Up and Go (TUG), Five-Times Sit-to-Stand (5 × STS), and 10-Meter Walk Test (10MWT). The 2-Minute Step Test required participants to step in place for two minutes, raising each knee to a point midway between the patella and iliac crest, with the number of right-knee raises recorded as an indicator of endurance [51]. For the TUG, participants stood from a seated position, walked three meters, turned around the cone, returned, and sat down; total time (seconds) was recorded, with shorter times reflecting better mobility [52]. The 5 × STS required participants to rise from a seated position and return to sitting five times as quickly as possible, with time recorded in seconds [53]. Gait speed was assessed using the 10MWT administered on a Zeno Walkway System (ProtoKinetics, Havertown, PA, USA), which captures temporal and spatial gait parameters through embedded pressure sensors located in a sensitive to pressure mat [54]. Participants were instructed to walk as fast as possible across a 10 m walkway, with the central six meters timed to exclude acceleration and deceleration phases. Gait speed (miles per hour; mph) and time to complete the 10-Meter Walk Test (seconds) were assessed, with higher gait speed values and lower completion times indicating better walking performance [55].

Balance was measured using the Berg Balance Scale (BBS) and postural sway analysis through APDM Opal wearable sensors. The BBS is a 14-item performance-based measure that assesses static and dynamic balance through common functional activities, including standing with eyes closed, turning 360 degrees, reaching forward, retrieving an object from the floor, and transferring between sitting and standing positions. Each item is scored from 0 (unable to perform) to 4 (performs independently), with a maximum total score of 56, where higher scores indicate better balance performance. The BBS has demonstrated excellent internal consistency (α > 0.90) and test–retest reliability (ICC = 0.98) in older adults and individuals with mobility disabilities [56,57,58].

Postural sway was assessed using APDM Opal inertial measurement units (IMUs) positioned at the level of lumbar area. Participants completed four quiet-standing conditions, each for 30 s: eyes open on firm surface, eyes closed on firm surface, eyes open on foam surface, and eyes closed on foam surface. The sway was quantified as total angle in degrees across the anterior–posterior, medial–lateral axes, as well as the whole area. Lower angles indicate less sway and better postural stability. The APDM system has demonstrated high reliability and validity for quantifying postural control in clinical and research settings [59].

Grip strength was assessed using a Jamar^®^ Smart Digital hand dynamometer (Patterson Medical, Warrenville, IL, USA). Three trials were completed per hand with approximately 30 s of rest between attempts, and the average value (kg) was recorded for the dominant and non-dominant hands. Participants were seated with the shoulder in a neutral position, elbow flexed to approximately 90°, and forearm in a neutral position. Grip strength assessment using these methods has demonstrated excellent test–retest reliability (ICC = 0.98) and criterion validity for upper extremity strength testing [60,61].

Health, Fitness, and Quality of Life Outcomes. Additional measures included height and weight for calculating body mass index (BMI), waist circumference, and fitness-related outcomes assessing changes in work capacity through three benchmark tests. BMI was derived from measured height and weight using standardized procedures. Body weight (kg) was measured twice using a calibrated digital scale (Befour PS-6600, Saukville, WI, USA), and height (cm) was measured twice using a portable stadiometer (Invicta IP-0955, Leicester, UK). BMI was calculated as weight (kg) divided by height (m^2^). Waist circumference was measured according to the standardized protocol described by Lohman et al. [62], with three readings taken and the final value recorded as the average of the two closest measurements.

Work capacity is defined as an individual’s ability to perform mechanical work across varied modalities, intensities, and time domains [63,64]. Although HIFT-based work capacity tests are not laboratory-based assessments, prior work in non-disabled populations has examined their relationship with traditional fitness indicators. Crawford et al. [63] demonstrated that changes in HIFT work capacity are strongly associated with changes in VO_2_max and ventilatory threshold, supporting their potential use as meaningful indicators of fitness in functional training contexts. Given the absence of established validity and reliability evidence for work capacity testing in adults with mobility disabilities, these outcomes were included in the present study as exploratory outcomes. Work capacity was assessed using three standardized HIFT workout tests, with each test administered on a separate day at both baseline and post-intervention. See Table 3 for descriptions of each work capacity test. Testing was conducted at participants’ selected training facilities by the site’s lead trainer, with loads, adaptations, and equipment settings (e.g., damper and station setup) documented to ensure consistency across sessions.

Finally, quality of life (QOL) was assessed using the World Health Organization Quality of Life–BREF (WHOQOL-BREF) instrument [65]. The WHOQOL-BREF is a 26-item self-report measure that evaluates perceived quality of life across four domains: physical health, psychological well-being, social relationships, and environmental context. Each item is rated on a 5-point Likert scale assessing intensity (*1 = not at all, 5 = an extreme amount*); frequency (*1 = never, 5 = always*), and capacity or satisfaction (*1 = very poor/dissatisfied, 5 = very good/satisfied*), with higher scores indicating better perceived quality of life. The WHOQOL-BREF has demonstrated strong internal consistency across domains (Cronbach’s α = 0.66–0.84) test–retest reliability, and construct validity in older adults and individuals with chronic or mobility-related disabilities [65].

### 2.5. Data Analysis

Quantitative data were analyzed in IBM SPSS Statistics (version 28, IBM Corp., Armonk, NY, USA) using descriptive statistics and effect sizes (Cohen’s *d*) to evaluate changes from baseline to post-intervention, as suggested for pilot and feasibility studies [37]. Cohen’s *d* was calculated as the mean change from baseline to post-intervention divided by the pooled standard deviation, where SD_pooled = √[(SD_pre^2^ + SD_post^2^)/2]; no small-sample correction was applied Effect size values of 0.2, 0.5, and 0.8 were interpreted as small, moderate, and large effects, respectively, and were used to describe the magnitude of change [66]. Participants with missing data were excluded from analysis for that measure. Given the small sample size, inferential statistics were not conducted nor were confidence intervals calculated, as the study was not designed for hypothesis testing and effect sizes were reported descriptively to inform feasibility. Exit interviews (~30–45 min) were conducted within 10 days of intervention completion by the study PI (Koon), who has experience conducting qualitative interviews in disability and aging research, and research assistant trained in qualitative methodology, audio-recorded, and transcribed verbatim. Any identifying information (e.g., the participant’s name) was removed from the final transcript. A high-level thematic analysis approach appropriate for feasibility pilots was applied [67,68]. Two researchers independently coded transcripts and reconciled discrepancies with a third reviewer to ensure that themes accurately reflected participants’ experiences and were informed by multiple perspectives.

## 3. Results

### 3.1. Participants

A total of 13 individuals provided informed consent to participate in the study. Of these, 10 participants completed the intervention and post-assessments, comprising the final analytic sample (Table 4). Participants ranged in age from 61 to 82 years (Mean age = 69.8, SD = 6.7) and were predominantly female (60%). Primary disability types included neurological conditions (Parkinson’s disease or stroke), injuries (traumatic injury), and health-related impairments (spinal stenosis). While participants were classified by a primary condition contributing to mobility impairment, functional limitations in the sample was multifactorial, reflecting the interaction of disability- and age-related changes. The time since disability onset varied widely across participants, ranging from 3.8 to 67.1 years, with an average duration of 16.5 years living with a mobility disability. Half of the participants reported using an assistive device for mobility at least some of the time.

### 3.2. Feasibility

**Figure 1:**
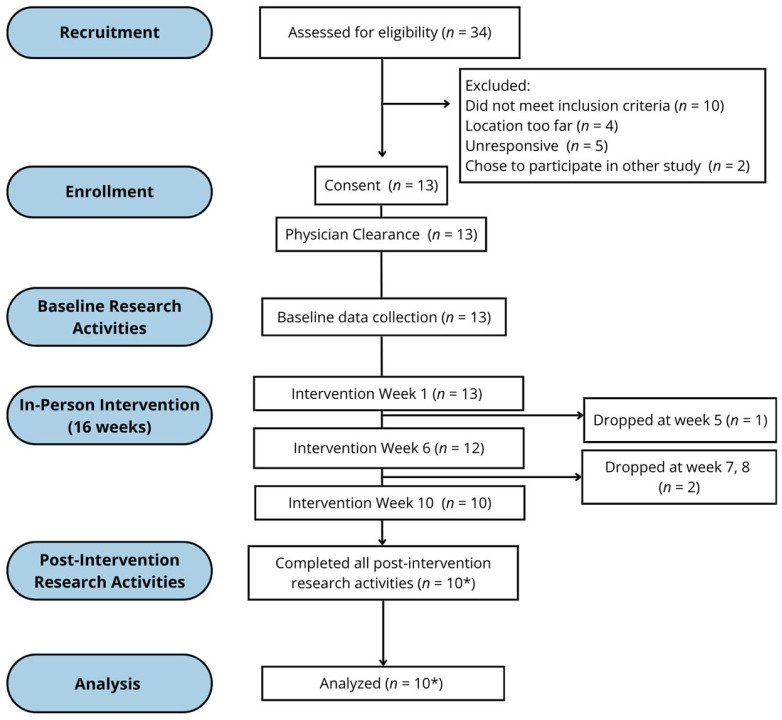
Participant flow through the study. * Baseline data collection and analyses varied by measure (range: 6–10 participants).

Figure 1 shows participant flow through the study. A total of 34 individuals completed the initial interest survey. Of these, 21 were ineligible or declined participation, while 13 participants provided informed consent and enrolled in the study, resulting in a 38% recruitment rate. Thirteen participants initiated the 16-week HIFT intervention, and 10 participants completed post-intervention assessments (retention rate = 77%). Three participants discontinued participation during the intervention. One participant withdrew following an injury that occurred during a training session (reported as an adverse event below), and two others withdrew due to unrelated health concerns and transportation issues. Attendance was calculated as both the total number of sessions attended out of 48 prescribed sessions and the average number of sessions attended per week. Participants attended an average of 28 sessions out of 48 prescribed sessions, resulting in an attendance rate of 58% across the 16 weeks. To better reflect real-world implementation, participants were permitted to make up missed sessions up to week 20. When this flexible timeframe was applied, attendance increased to 80% (38 sessions out of 48 prescribed). On average, participants attended 2.4 sessions per week regardless of timing of intervention completion. Compared with the predefined feasibility thresholds (≥70% recruitment, ≥80% retention, ≥75% attendance) [36,37]. retention approached the targeted benchmark, whereas recruitment and session-based attendance during the 16-week intervention fell below the intended thresholds. Session-level perceived exertion was monitored using the Borg CR-10 scale [31]. Across 298 recorded sessions, participants reported a mean RPE of 6.2 ± 1.4 (range 3.9–8.2), indicating moderate to vigorous perceived effort during HIFT sessions.

Semi-structured exit interviews provided additional context regarding intervention acceptability. Participants consistently described the intervention as appropriately individualized. The accessible equipment and proactive adaptations provided by the trainers were central to participants’ confidence and ability to engage in the sessions safely and effectively. As one participant described, *“They knew everybody’s limitations and fixed things right away, so I didn’t hurt myself.”* Participants commonly reported improvements in everyday functional activities—including walking longer distances, stair negotiation, household chores, and gardening: *“I’m just stronger. I’m able to pick things up easier, like off the ground”*. Psychosocial benefits were also described, especially the supportive, peer-based group environment. One participant shared, *“I just love being with the people. I don’t have many places where I get to be around others with disabilities”*, while another stated, *“It feels really good to be with people who understand what [having a disability is] like”*. Barriers to attendance were described primarily as logistical (illness, weather, transportation, work/caregiving responsibilities).

During the intervention, one participant fell while transitioning from a bench to a standing position. Trainers responded immediately and provided appropriate assistance; the participant did not complete the remainder of that session. The participant subsequently missed approximately four weeks of classes due to the injuries sustained from the fall and ultimately withdrew from the study at week eight. The other two adverse events were unrelated to the intervention and involved a pre-existing overuse injury that flared early in the study (week five) and an illness of a spouse that affected transportation (week seven).

### 3.3. Physical Function

Participants reported improvements completing daily activities they deemed important, with large, positive effects observed for both performance (*d* = 1.03) and satisfaction with performance (*d* = 1.54). During the COPM interviews, participants identified a range of activities across self-care, productivity, and leisure domains including getting dressed, using the toilet, climbing stairs, showering, and getting in and out of bed (self-care); cleaning the floor, preparing meals, carrying laundry upstairs and downstairs, and vacuuming (productivity); and playing adaptive sports, gardening, playing the drums, and going out with friends (leisure). A small effect was observed on the PROMIS Physical Function CAT (*d* = 0.36), reflecting modest change in self-reported ability to perform daily and mobility-related activities. A large effect was observed for confidence performing daily activities without falling, as measured by the Modified Falls Efficacy Scale (*d* = 0.97), indicating higher fall-related self-efficacy post-intervention.

Performance-based measures of functional mobility demonstrated variable effects across domains. Performance on the 2-Minute Step Test showed a large effect (*d* = 1.01), with participants completing an average of 10.6 more steps post-intervention. In contrast, mobility outcomes demonstrated small declines, with participants taking slightly longer to complete the 10-Meter Walk (*d* = 0.25) and demonstrating a small decrease in average walking speed (*d* = −0.18). Minimal change was observed for the Timed Up and Go (*d* = 0.08), along with a small effect for the Five-Times Sit-to-Stand (*d* = −0.29). Balance outcomes demonstrated modest change across measures. Participants’ BBS scores increased by an average of 2.3 points with moderate effects (*d* = 0.55). Small changes were also noted in postural sway measures across all four conditions (*d* = 0.17–0.52), suggesting overall maintenance of static and dynamic balance, and postural stability throughout the intervention. Only six participants completed the most challenging condition (eyes closed on foam surface), which may have limited the precision of change estimates for this test.

### 3.4. Health, Fitness, and Quality of Life

Performance on all three work capacity tests improved following the 16-week intervention. Two participants did not complete post-testing, resulting in an analytic sample of eight. The 10 min AMRAP showed a moderate effect (*d* = 0.61), while the 20 min AMRAP demonstrated a large effect (*d* = 1.43). Performance on the 250 m row improved substantially, with participants completing the task an average of 13.8 s faster post-intervention, reflecting a large effect (*d* = −1.20). Body mass index and waist circumference remained stable across the intervention (BMI *d* = 0.05; waist circumference *d* = 0.02). Finally, overall quality of life demonstrated a small favorable change, with WHOQOL-BREF scores showing a small effect (*d* = 0.20).

A comprehensive summary of outcome variables, including sample size, pre- and post-intervention means and standard deviations, mean change scores, and Cohen’s *d* effect sizes, is presented in Table 5.

## 4. Discussion

Feasibility and Implementation. This pilot study demonstrated that a community-based HIFT program is feasible, safe, and acceptable for older adults with mobility disabilities. Retention was strong, with 77% of enrolled participants completing post-intervention assessments, approaching predefined feasibility benchmarks. In contrast, recruitment fell below target thresholds, likely reflecting the use of strict inclusion criteria that required participants to have a mobility disability while remaining ambulatory. Average weekly attendance was also below the intended target of three sessions per week; however, participants engaged consistently, attending an average of 2.4 sessions per week and completing 80% of prescribed sessions when flexible scheduling was permitted. This level of participation meets current physical activity recommendations for muscle-strengthening activity at least two days per week for disabled [69] and non-disabled adult populations [70]. Prior studies of community-based HIFT among older adults without disabilities have reported higher attendance (87.5%) and retention (100%) [29], while studies involving people with mobility disabilities have shown similarly strong engagement despite more complex functional needs [26,27,28]. The adherence patterns observed in the current study align with prior community-based disability exercise studies, in which session completion commonly ranges around 60% due to fluctuating health, transportation challenges, and caregiving responsibilities [71,72]. Findings from the current study suggest that prescribing three sessions per week may exceed what is feasible for some older adults aging with mobility disabilities, highlighting the need to evaluate alternative dosing models in future trials. Together, these findings reinforce that adaptive scheduling and real-world implementation support are essential for sustained participation among older adults with mobility disabilities.

Self-Report and Performance-Based Functional Health Implications. Participant reported measures of performance and satisfaction as well as fall-related self-efficacy demonstrated the largest changes, while standardized self-reported physical function showed smaller effects. Although large improvements in COPM outcomes suggest that participation translated into enhanced ability to complete activities deemed most important by participants, these effect size estimates should be interpreted conservatively given the pilot nature of the study and limited sample size, as estimates of magnitude may be unstable and associated with relatively wide confidence intervals. Qualitative data supported these findings, as participants described improved strength and balance, greater independence and confidence in performing daily activities, and reduced fear of injury. the results also align with previous pilot trials exploring the effects of HIFT for people with mobility disabilities [28], qualitative work showing that HIFT supports various aspects of physical function for individuals with mobility-related disabilities [73]. These findings are especially relevant since maintaining or improving function is consistently identified as a primary reason why individuals with mobility disabilities choose to exercise [74,75].

Performance-based physical function outcomes were variable across domains. For example, substantial gains in endurance via the 2-Minute Step Test were observed, while minimal changes in measures of mobility (10 m walk) and strength (TUG, 5 × STS) were recorded. Results from balance assessments were also mixed. Participants showed modest improvements on the Berg Balance Scale, whereas postural sway values were largely maintained across standing conditions, with only slight improvements observed in the most challenging task (eyes closed on foam), though interpretation is limited by small completion numbers. Importantly, maintenance of balance may represent a favorable outcome, given high baseline balance performance and the expected age-related trajectory of balance decline among adults aging with mobility disabilities. Previous HIFT trials involving adults with mobility disabilities have generally reported consistent improvements across performance-based functional outcomes. For example, Handlery et al. [26] demonstrated clinically meaningful gains in endurance, strength and balance following a 25-week community-based HIFT program for adults with spinal cord injury. Likewise, Koon et al. [28] observed large performance-based improvements in a 24-week adaptive HIFT trial for adults with mobility disabilities and overweight/obesity, including large, positive effects on strength (*d* = 1.01), endurance (*d* = 1.28), and speed (*d* = –0.95). Among older adults without disabilities, Heinrich et al. [29] reported small but consistent improvements across standardized mobility and strength assessments (*d* = 0.20–0.45) following an 8-week HIFT program, though only one test reached statistical significance. Further, Handlery et al. [27] found that older adults with Parkinson’s disease demonstrated moderate improvements in endurance, gait speed, and balance (r = 0.53–0.63) following a 25-week HIFT program.

The current and prior studies suggest that HIFT can reliably preserve and, in some cases, improve performance-based functional outcomes across diverse disability and aging groups; however, the magnitude of change appears to vary by age, baseline mobility level, and intervention duration. In the present study, participants entered the intervention with relatively high baseline functional capacity on standardized mobility measures, which may have influenced change due to ceiling effects and contributed to more modest performance-based outcomes. In contrast, self-reported functional measures may be more sensitive to functional changes, particularly those involving task-based activities deemed important by the participant. Together, these findings suggest that variability in responsiveness across outcomes likely reflect differences in baseline function and measurement sensitivity, rather than inconsistency in intervention effectiveness.

In contrast to the variability observed across traditional mobility and strength assessments, work capacity outcomes demonstrated moderate to large improvements across all three benchmark tests. These findings suggest that the functional and multimodal approach from HIFT programming may support improvements in overall fitness that are not fully reflected in isolated strength or mobility tests. The magnitude of work capacity gains in the present study parallels those observed previous HIFT pilots, in which adults with mobility disabilities showed moderate to large improvements (*d* = 0.65–1.59) across two work capacity tests following a 12-week HIFT intervention [28] and adults with mobility disabilities and overweight/obesity demonstrated large improvements across three work-capacity tests following a 24-week intervention (*d* = 0.97–1.39) [28]. Although the current study used three standardized workouts to capture changes in fitness, the field would benefit from developing and validating a unified work-capacity testing framework for adults with mobility disabilities.

Changes to QOL were small in magnitude in the present study; however, the positive direction of change is consistent with earlier disability-focused HIFT trials. In the 24-week study, adults with mobility disabilities and overweight/obesity demonstrated similarly small effects in QOL (*d* = 0.25), whereas a 12-week HIFT program among adults with mobility-related disabilities reported large effects in QOL (*d* = 1.04) [28]. Qualitative feedback from the current study further supports this interpretation, as participants described the group-based format and supportive relationship with training staff as central to their overall experience.

Sustainability and Real-World Implementation. The present trial was embedded in an existing community-based HIFT program rather than in a clinical or university setting. Thus, participants were able to continue attending HIFT classes after the funded intervention period ended. Four weeks post-intervention, 60% of participants were still attending classes, with 50% still attending sessions 16 weeks post intervention. The sustained participation patterns mirror follow-up findings from previous adaptive HIFT trials [28,76], in which most of the research participants elected to continue independently after study completion, underscoring the value of an inclusive and accessible community-based exercise model. Future studies should extend this work through larger, multi-site trials with diverse disability groups to evaluate effectiveness and real-world implementation across different community settings, including systematic assessments of trainer fidelity, adaptation procedures, and organizational readiness. Intervention designs that incorporate additional support, such as transportation assistance, or remote session options, may help address the fluctuating health and environmental barriers. Finally, research should explore whether improvements in work capacity translate to meaningful changes in daily activities, participation, and quality of life, thereby strengthening the evidence base for HIFT as a resource for community-based exercise.

Strengths and Practical Contributions. Strengths of this study include delivery of the intervention within existing community-based fitness facilities, use of certified trainers with expertise in adaptive and inclusive training, and emphasis on individualized movement modifications to support engagement. The mixed-methods design, integrating feasibility metrics, self-reported outcomes, performance-based assessments, and qualitative exit interviews, provides a comprehensive evaluation of intervention feasibility, initial effectiveness, and participant experience. Embedding the program in real-world settings enhances ecological validity and supports the potential scalability of HIFT for adults aging with mobility disabilities.

Limitations. Several limitations should be considered when interpreting the findings. As a single-group pre–post feasibility pilot with a small sample, the study was not designed for causal inference, and effect size estimates should be interpreted with caution given limited precision, potential instability, and restricted generalizability. Accordingly, observed effects should be viewed as preliminary indicators intended to inform feasibility and future hypothesis testing rather than definitive intervention effects.

Participants were required to be ambulatory and, on average, demonstrated relatively high performance-based function at baseline, which may have contributed to ceiling effects on certain performance-based assessments and partially explains the maintenance observed across functional outcomes. Furthermore, inclusion criteria likely contributed to a more motivated sample, limiting generalizability to the broader and more diverse population of older adults with mobility disabilities. Future trials may benefit from broader inclusion criteria and expanded recruitment strategies to enhance enrollment and sample diversity.

The prescribed three-session-per-week training dose may not represent a realistic or optimal participation target for all individuals in this population, and alternative dosing strategies should be explored in future studies. Additionally, qualitative data were coded by the lead investigator, which may introduce potential confirmation bias; future studies should incorporate independent coding or auditing procedures to enhance analytic rigor. Finally, fall-related self-efficacy was assessed using the MFES; however, objective fall risk or fall incidence measures were not collected and should be considered in future trials.

## 5. Conclusions

This pilot study provides preliminary evidence that a community-based HIFT program is a feasible and acceptable exercise model for adults aging with mobility disabilities. Participants reported meaningful improvements in perceived functional ability, while changes in objective physical performance were mixed and generally modest, underscoring the need for adequately powered controlled trials to establish effectiveness. Overall, the current findings support community-delivered HIFT as a promising and scalable approach for improving exercise access and perceived function in this population.

## Figures and Tables

**Table 1:** Sample HIFT workout of the day (WODs) with seated and standing adaptations.

Session Type	Standing	Seated
**As many rounds as possible (AMRAP)**	10 min AMRAP: 10 Burpees; 5 squats; 10 push-ups; 5 dips	10 min AMRAP: 10 slam balls; 5 dips; 5 shoulder presses; 3 dips
**Rounds for Time (RFT)**	3 RFT: 20 squats; 20 sit-ups; 20 shoulder press; 20 kettlebell swings	3 RFT: 12 dips; 40 weighted twists; 20 dumbbell shoulder press; 20 dumbbell front raise
**Every minute on the minute (EMOM)**	12 min Total: Minute 1: 18 alternating dumbbell row; Minute 2: 200 m bike; Minute 3: 9 deadlifts; Minute 4: rest (repeat for rest of time)	12 min Total: Minute 1: 18 alternating dumbbell row; Minute 2: 200 m bike; Minute 3: 10 side-to-side deadlifts; Minute 4: rest (repeat for rest of time)

Note. AMRAP = As Many Rounds As Possible; RFT = Rounds for Time; EMOM = Every Minute on the Minute.

**Table 2:** Example trainer log used for intervention fidelity monitoring. Rows reflect fields completed by trainers for each participant following a HIFT session.

26-Aug	Participant A: (19/48 Sessions)	Participant B: (30/48 Sessions)
**Location/Time**	Mission, 10:30 a.m.	NKC, 3:30 p.m.
**Week of Intervention**	8	12
**Adapted WOD**	Standing	Seated
**RPE**	4	7
**Personal Adaptation Notes**	AMRAP 8: shuttle runs + deadlifts; 4 min rest; AMRAP 8: shuttle runs + push-ups.	AMRAP 8 min: shuttle rolls + landmine rows; 4 min rest; AMRAP 8 min: shuttle rolls + landmine press.
**PPT Results**	6 rounds + 3 reps	9 rounds + 7 reps
**Adverse Events**	None	None
**Additional Notes**	Reported fatigue from previous night’s sleep	__

Note. WOD = workout of the day; RPE = Rating of Perceived Exertion (Borg CR-10); PPT = participant; reps = repetitions; AMRAP = As Many Rounds As Possible; min = minute. Table presents an example of the trainer-completed session log used to document adaptations, perceived exertion, performance, and safety during intervention delivery.

**Table 3:** Work Capacity Test Details.

Work Capacity	Description	Standardized Test Condition	Outcome Measured	Functional Relevance
10 min AMRAP	Perform as many rounds + repetitions as possible: 9 Calorie Row + 15 Dumbbell Clean and Press (standing); 6 Calorie Row + 15 Ground-to-Overhead Plate (seated)	Rower damper setting Load used (DBs or plate) Rower serial #	Total rounds + repetitions completed in 10 min	Lifting/carrying under fatigue Multi-joint movement integration
20 min AMRAP	Perform as many rounds + repetitions as possible: 5 Calorie Bike + 7 Push-ups + 9 Box Squats (standing); 2 Dips + 3 Calorie Arms-Only Bike + 8 Side-to-Side Deadlifts with Kettlebell (seated)	Push-up/dip bar height Box height/ Kettlebell weight Bike serial # Station setup for transition time	Total rounds and repetitions completed in 20 min	Transfers, toileting, lap-floor reach Total-body endurance in functional tasks
For Time	Complete: 250 m Row (standing); 180 m Ski (seated)	Damper setting Row/Ski Erg serial # Consistent warm-up protocol	Time to completion	High-output, rapid work tasks (e.g., emergency transfers, sprint-carry, quick response in daily activities)

Note. AMRAP = As Many Rounds As Possible; DB = Dumbbell; # = number.

**Table 4:** Participant demographics and descriptives (*N* = 10).

Variable	Category	*n* (%)
Sex	Female	6 (60%)
	Male	4 (40%)
Age	61–66	4 (40%)
	67–73	4 (40%)
	74–79	1 (10%)
	80+	1 (10%)
Education	1–3 years of college	1 (10%)
	College degree or more	9 (90%)
Race	White	9 (90%)
	American Indian/Alaskan Native	1 (10%)
Hispanic/ Latino	No	10 (100%)
Marital Status	Married	7 (70%)
	Divorced	2 (20%)
	Widowed	1 (10%)
Income	<$50,000	2 (20%)
	$50,000–$100,000	6 (60%)
	>$100,000	1 (10%)
	No response	1 (10%)
Disability Type	Neurological (e.g., Parkinsons, Stroke)	3 (30%)
	Health Impairment (e.g., Rheumatoid Arthritis, Stenosis)	4 (40%)
	Injury (e.g., Traumatic Brain Injury)	1 (10%)
	Other (e.g., Essential Tremor, Osteoarthritis)	2 (20%)
Time since onset of disability	<10 years	5 (50%)
	10–29 years	4 (40)
	≥30 years	1 (10%)
Assistive Devices	Yes, utilizes assistive devices (e.g., cane, grab bars, orthotic device, walker, grabber)	5 (50%)
	No, does not utilize assistive devices	5 (50%)

**Table 5:** Baseline and post-intervention means, standard deviations, change scores and effect sizes.

Outcome	N	Baseline M (SD)	Post M (SD)	Δ M (SD)	Cohen’s *d*	Interpretation
Self-Report Physical Function						
COPM—Performance ^a^	10	4.6 (1.33)	6.2 (1.84)	1.7 (1.24)	1.03 *	Large
COPM—Satisfaction ^a^	10	3.7 (1.14)	6.2 (2.01)	2.5 (1.87)	1.54 *	Large
PROMIS Physical Function ^b^ (T-score)	10	39.3 (3.97)	40.9 (4.76)	1.6 (3.14)	0.36	Small
Modified Falls Efficacy Scale ^c^ (MFES)	10	6.8 (1.8)	8.4 (1.5)	1.6 (1.8)	0.97 *	Large
Performance-Based Physical Function						
2-Minute Step Test ^d^	9	68.7 (10.3)	79.2 (10.6)	10.6 (9.1)	1.01 *	Large
(Number of steps)						
Timed Up and Go ^e^ (sec)	10	7.5 (1.5)	7.7 (3.1)	0.2 (2.4)	0.08	Small
Five-Times Sit-to-Stand ^e^ (sec)	9	10.1 (2.9)	9.29 (2.6)	−0.8 (1.9)	−0.29	Small
10-Meter Walk ^f^ (sec)	10	7.4 (1.7)	7.9 (2.3)	0.5 (1.0)	0.25	Small
10-Meter Walk ^f^ (mph)	10	3.7 (1.1)	3.5 (1.1)	−0.2 (0.5)	−0.18	Small
Berg Balance Scale ^g^	10	50.8 (4.0)	53.1 (4.4)	2.3 (5.1)	0.55	Moderate
Postural Sway ^h^ (°)—eyes open, firm surface	10	1.1 (0.8)	1.6 (1.1)	0.5 (1.6)	0.52	Small
Postural Sway ^h^ (°)—eyes open, foam surface	10	5.0(4.0)	5.6 (4.3)	0.7 (3.2)	0.17	Small
Postural Sway ^h^ (°)—eyes closed, firm surface	10	1.9 (1.2)	2.3 (1.2)	0.4 (1.8)	0.33	Small
Postural Sway ^h^ (°)—eyes closed, foam surface	6	21.9 (10.8)	14.5 (7.0)	−7.4 (15.9)	−0.81	Small
Grip Strength (kg)—dominant hand	10	26.8 (8.8)	28.1 (7.4)	1.2 (6.2)	0.16	Small
Grip Strength ^i^ (kg)—non-dominant hand	10	25.8 (9.8)	26.9 (8.8)	1.17 (5.7)	0.12	Small
Health, Fitness, Quality of Life						
BMI ^j^ (kg/m^2^)	10	26.0 (3.7)	26.2 (3.7)	0.1 (0.8)	0.05	Small
Waist-circumference ^k^	9	86.8 (12.8)	87.1 (16.2)	0.4 (5.1)	0.02	Small
Work Capacity ^l^ 10 min AMRAP (rounds + repetitions)	8	3.3 (1.1)	4.1 (1.5)	0.7 (0.7)	0.61	Moderate
Work Capacity ^l^ 20 min AMRAP (rounds + repetitions)	8	6.6 (1.8)	9.8 (2.6)	3.2 (1.2)	1.43 *	Large
Work Capacity ^m^ 250 m row (sec)	8	98.0 (8.9)	84.2 (13.6)	−13.8 (15.6)	−1.20 *	Large
WHOQOL-BREF ^n^ (Overall)		3.8 (0.5)	3.9 (0.5)	0.1 (0.2)	0.20	Small

Note. All values represent group means and standard deviations (SD). N reflects the number of participants with complete baseline and post-intervention data for each measure; sample sizes vary due to missing assessments and measure-specific feasibility constraints. Effect sizes were interpreted using Cohen’s d, where 0.20 = small, 0.50 = moderate, and 0.80 = large. Δ = mean change from baseline to post-intervention; positive values indicate improvement unless otherwise noted. * = large effect size. ^a^ COPM Performance and Satisfaction scores range from 1–10; higher scores indicate greater perceived performance/satisfaction. ^b^ PROMIS Physical Function reported as standardized T-scores (mean = 50, SD = 10); higher scores indicate greater perceived physical function. ^c^ Modified Falls Efficacy Scale (MFES) scores range from 0–10; higher scores indicate greater balance confidence. ^d^ 2-Minute Step Test reported as total number of steps completed in 2 min; higher values indicate greater endurance. ^e^ Timed Up and Go (TUG) and Five-Times Sit-to-Stand (5 × STS) measured in seconds; lower times indicate better performance. ^f^ 10-Meter Walk Test reported as time to complete the walk (seconds) and gait speed (miles per hour; mph); lower completion times and higher gait speed values indicate better walking performance. ^g^ Berg Balance Scale (BBS) scores range from 0–56; higher scores indicate better balance. ^h^ Postural sway values reflect mean degrees of sway (°); lower values indicate better postural stability. ^i^ Grip strength measured in kilograms (kg) using a standardized hand-held dynamometer; higher scores indicate greater strength. ^j^ Body Mass Index (BMI) was calculated as weight (kg) ÷ height (m^2^). ^k^ Waist circumference measured to the nearest 0.1 cm at the umbilicus level. ^l^ Work capacity AMRAP = “as many rounds/repetitions as possible” within fixed time domains; higher values reflect greater work capacity. ^m^ Work capacity 250-m row “for time” (seconds); lower scores reflect improved work capacity; ^n^ World Health Organization Quality of Life BREF Version (WHOQOL-BREF) overall quality-of-life scores range from 1–5; higher scores indicate better perceived quality of life.

## Data Availability

The data presented in this study are available on reasonable request from the corresponding author. The data are not publicly available due to privacy and ethical restrictions.
